# Assessing Cardiac Mechanical Dysfunction in Transfusion‐Dependent β‐Thalassemia With History of Atrial Fibrillation: The Role of Speckle Tracking Echocardiography

**DOI:** 10.1111/echo.70408

**Published:** 2026-02-11

**Authors:** Federico Marchini, Maria Mele, Elena Marchetti, Laura Rotondo, Federica Frascaro, Michele Malagù, Rita Pavasini, Elisabetta Tonet, Matteo Serenelli, Alberto Cossu, Filomena Longo, Maria Lo Monaco, Erika Bertella, Gianluca Campo, Matteo Bertini

**Affiliations:** ^1^ Cardiology Unit Azienda Ospedaliero Universitaria Di Ferrara Ferrara Italy; ^2^ Division of Radiology Azienda Ospedaliero Universitaria Di Ferrara Ferrara Italy; ^3^ Thalassemia and Hemoglobinopathies Unit Azienda Ospedaliero Universitaria Di Ferrara Ferrara Italy; ^4^ Cardiovascular Institute Ospedale Humanitas Gavazzeni Bergamo Italy

**Keywords:** atrial fibrillation, global longitudinal strain, myocardial work, peak atrial longitudinal strain, speckle tracking echocardiography, transfusion dependent beta thalassemia

## Abstract

**Purpose:**

Atrial fibrillation (AF) is highly prevalent in patients with transfusion‐dependent beta‐thalassemia (TDT). Speckle tracking echocardiography (STE) provides detailed information about left ventricular (LV) and atrial function, however its role in TDT patients with AF has not been completely investigated. This study aimed to assess differences in cardiac mechanical parameters between thalassemia patients with and without history of AF.

**Methods and results:**

223 TDT patients in sinus rhythm were enrolled and, among them, 26 (11%) had history of AF. A complete STE analysis with the evaluation of global longitudinal strain (GLS), peak atrial longitudinal strain (PALS) and myocardial work (MW) indices were performed. The primary endpoint was the difference in STE parameters. The secondary endpoint was the prevalence of cardiac mechanical dysfunction. Patients with history of AF showed significantly lower values of GLS (19% vs 21%, *p* = 0.01) and PALS (24% vs 35%, *p* < 0.001) compared to those without AF. AF patients showed higher prevalence of both ventricular and atrial mechanical dysfunction (respectively 27% vs 12%, *p* = 0.03 and 42% vs 11%, *p* < 0.001). PALS showed high discriminative ability (AUC 0.76, 95% CI 0.66–0.85) with an optimal cut‐off value of 25.9% to detect those with history of AF.

**Conclusions:**

Among TDT patients, those with history of AF showed lower values of GLS and PALS. Both LV and atrial mechanical dysfunction were significantly prevalent in patients with history of AF and PALS showed high diagnostic accuracy for the detection of AF.

**Clinical trial registration:**

ClinicalTrials.gov id NCT05508932

## Graphical abstract

Transfusion dependent β‐thalassemia patients with history of atrial fibrillation (AF) had lower values of global longitudinal strain and peak atrial longitudinal strain (PALS) and showed higher prevalence of both left ventricular and atrial mechanical dysfunction. PALS was associated with high diagnostic accuracy for the detection of AF.

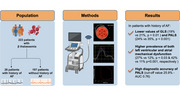

## 1 Introduction

Transfusion dependent β‐thalassemia (TDT) is an inherited blood disorder characterized by reduced or absent production of β‐globin chains of haemoglobin and chronic anaemia of varying severity [1]. In developed countries, the prognosis of patients with TDT has significantly improved thanks to the introduction of blood transfusions and iron chelation therapy, which helped prevent several life‐threatening complications such as heart failure and malignant arrhythmias [2, 3].

Nowadays, in this cohort of patients, atrial fibrillation (AF) represents the most common arrhythmic disorder [4]. The reported incidence of AF in TDT is heterogeneous among the studies, ranging from 2% to 33% [5]. Its pathophysiology is complex and multifactorial, involving anaemia and frequent blood transfusions, which lead to iron overload and cardiomyocytes infiltration that affects cardiac electrical conduction; moreover, atrial function may be compromised by volume overload and hyperdynamic circulation, leading to atrial dilatation and structural remodelling, potentially preceding the onset of iron overload [6].

Speckle tracking echocardiography (STE) with evaluation of global longitudinal strain (GLS) can be integrated with 2D or 3D echocardiographic techniques to analyse the complex directional components of left ventricular (LV) deformation [7]. Myocardial work (MW), an advanced application of speckle tracking analysis, has proven to be a useful method for evaluating myocardial systolic performance by integrating deformation parameters with loading conditions [8]. Recently, left atrial deformation analysis by 2D STE, focusing on the assessment of peak atrial longitudinal strain (PALS), has gained clinical importance, allowing for comprehensive analysis of left atrial function and possibly detecting subclinical dysfunction even before structural changes occur [9].

The role of STE in TDT patients with history of AF has not been completely investigated. This study aimed to explore the differences in cardiac mechanics and the prevalence of mechanical dysfunction between TDT patients with and without history of AF.

## 2 Materials and Methods

AF in β‐thalassemia *“*β‐THAL” is a prospective, single‐centre, observational study conducted at the Azienda Ospedaliero‐Universitaria di Ferrara, Italy. The study collected clinical, laboratory, electrocardiographic and imaging characteristics in patients with TDT and was registered on ClinicalTrials.gov (NCT05508932). Study protocol was approved by the local Ethics Committee (Comitato Etico Indipendente di Area Vasta Emilia Centro). The study was conducted in accordance with the ethical principles of the Declaration of Helsinki and all patients signed informed consent.

### 2.1 Study Population

Inclusion criteria were confirmed TDT, age ≥ 18 years and sinus rhythm during echocardiography. Exclusion criteria were state of pregnancy, inability to give informed consent, AF rhythm during echocardiographic examination and low quality of echocardiographic images that do not allow the execution of STE. As part of their standard care, patients received yearly routine cardiology evaluations, echocardiographic examination and cardiac magnetic resonance imaging (CMR). Participant management was at the discretion of the attending physicians and followed institutional protocols and international guidelines.

### 2.2 Echocardiographic Examination

A comprehensive echocardiographic, Doppler and Color Doppler examination was performed using a GE Vivid E80, S60 or E9 echo scanner (GE Health Care, Milwaukee, WI) equipped with a 3.5 MHz transducer. Echocardiographic images were stored in digital format and analysed using the EchoPAC software v. 202 (GE Health Care, Milwaukee, WI). Two expert trained cardiologists (F.M and M.M.) did all the echocardiographic measures, according to the American Society of Echocardiography/European Association of Cardiovascular (CV) Imaging guidelines [10, 11]. Image and Doppler acquisitions were obtained at held end‐expiration. During apnoea, two cardiac cycles were recorded for every image and then stored in cine‐loop format with a frame rate between 40 and 80 Hz. Non‐invasive measurement of systolic and diastolic blood pressure has been performed during the exam by measurement in the arm using a sphygmomanometer. GLS, PALS and indices of MW (Global work index (GWI), global constructive work (GCW), global wasted work (GWW) and global work efficiency (GWE)) were obtained as previously reported [12]. Normal values were derived from published literature [9, 13]. GLS was reported as absolute values, with lower values indicating worse LV longitudinal systolic function. The detailed echocardiography protocol is available on Supplementary Data.

### 2.3 Data Collection

Clinical, therapeutic, and outcome data were prospectively recorded through a dedicated electronic case report form (eCRF). All procedures were conducted by trained and qualified personnel. The eCRFs were regularly monitored and validated against source documentation. Retrospectively retrieved data included anthropometric measurements, CV risk factors, CV history and comorbidities, laboratory parameters, last available transthoracic echocardiography and CMR parameters. These data were extracted from patients' clinical records.

### 2.4 Study Endpoints

The primary endpoint was the difference in STE indices between TDT patients with or without history of AF. The secondary endpoint was the prevalence of cardiac mechanical dysfunction, defined as the reduction in STE indices, between patients with or without history of AF.

### 2.5 Statistical Analysis

Continuous data were tested for normal distribution with the Kolmogorov—Smirnov test. Normally distributed values were presented as mean ± SD and compared to a t‐test. Otherwise, median, interquartile range, and Mann‐Whitney U test were applied. Categorical variables were summarized in terms of counts and percentages and were compared by using the two‐sided chi‐square or Fisher's exact test. ROC curve analysis was conducted to evaluate the discriminatory power of STE indices in identifying patients with history of AF. To determine the optimal threshold, the Youden index method was applied. Univariate logistic regression models were used to calculate odds ratios and corresponding 95% confidence intervals (CIs) evaluating the relationship between each index of cardiac mechanics and history of AF. Clinical variables with a *p* value of < 0.1 in the univariate analysis were examined in the stepwise multivariate analysis. The stepwise models added or removed variables from this reduced list based on a significance level of 5% (*p*‐value < 0.05). A final multivariable model was then developed using the statistically significant independent variables identified by the stepwise model. To avoid collinearity, two multivariate models have been provided: the first model included age, pulmonary artery systolic pressure (sPASP), left atrial end systolic volume (LA ESV) indexed and GLS under the optimal cut point; the second model included age, sPASP, LA ESV indexed and PALS under the optimal cut point. The intra‐ and interobserver agreement between operators was tested with Bland Altman scatter plot analysis and intra‐class correlation coefficients on the first 10 TTEs analysed, showing good reproducibility. All statistical analyses were performed with Stata/SE version 16 software (Stata Corp, College Station, TX, USA).

## 3 Results

### 3.1 Baseline Characteristics of the Patients

**FIGURE 1:**
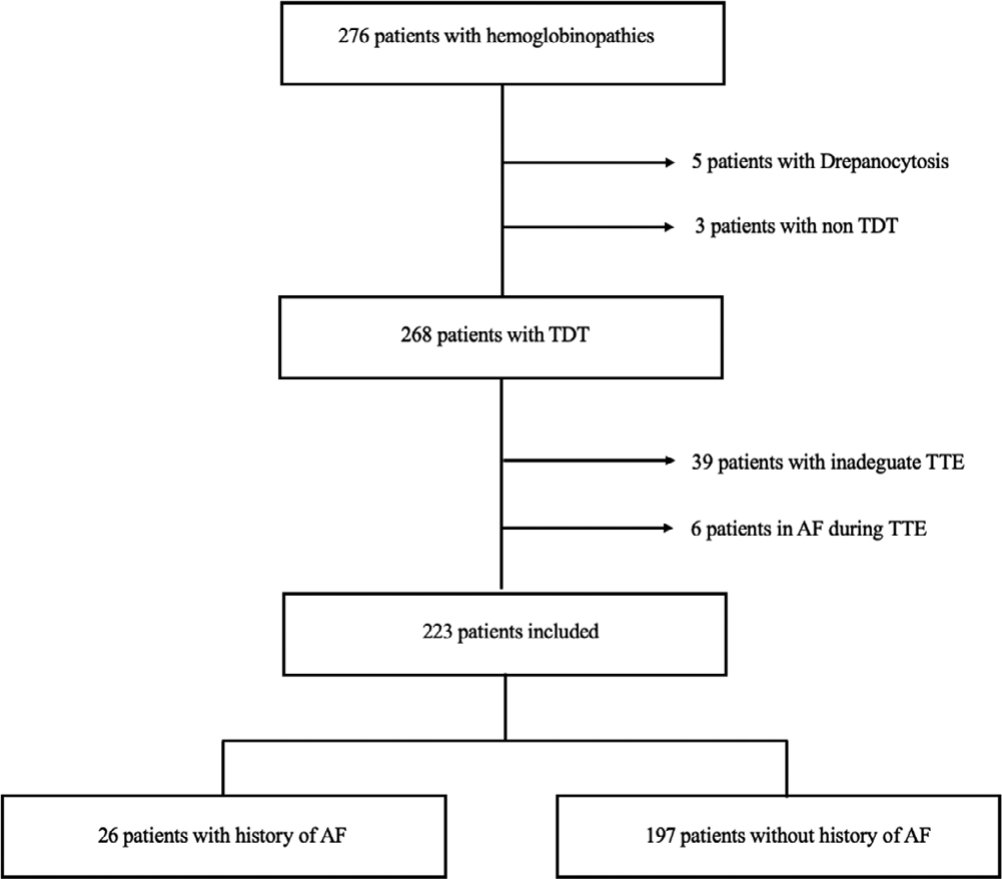
Flow chart of the study protocol. TDT: transfusion‐dependent β‐thalassemia; TTE: transthoracic echocardiography; AF: atrial fibrillation.

Between August 2022 and January 2025, 276 patients suffering from hemoglobinopathies were evaluated. Five patients with drepanocytosis and three patients with non‐TDT were excluded. 39 patients had echocardiographic images of insufficient quality to perform STE and MW and were also excluded. 6 patients were not included because of AF rhythm during the echocardiographic examination. Finally, 223 patients were enrolled in the present analyses. Among them, 26 patients (11%) had history of AF (Figure 1).

Overall, the median age was 50 years (IQR 45–55). Patients with history of AF were older (53 years old vs 50 years old, *p* = 0.04) and had a higher rate of pulmonary hypertension (6 patients vs 3 patients, *p* < 0.001). The use of diuretics, ace‐inhibitors or angiotensin receptor blockers, oral anticoagulants and antiarrhythmic drugs was more frequent in AF patients. Regarding echocardiographic characteristics, patients with history of AF had higher LA ESV indexed (41 mL/m2 vs 29 mL/m2, *p* < 0.001) and right atrial volume indexed (30 mL/m2 vs 24 mL/m2, *p* < 0.001), lower values of LV ejection fraction (LVEF) (59% vs 62%, *p* = 0.01) and higher values of sPASP (30 mmHg vs 25 mmHg, *p* = 0.001). With reference to CMR parameters, patients with history of AF had higher values of LV end diastolic mass (56 g/m2 vs 49 g/m2, *p* = 0.01). No differences were found in cardiac or liver T2* and T1 mapping values. Baseline characteristics of the patients are summarized in Table 1.

**TABLE 1:** Baseline characteristics of the patients.

	Total	History of AF	No AF	
	*N* = 223	*N* = 26	*N* = 197	*p*‐value
**Clinical characteristics**				
Age, yrs	50 (45–55)	53 (48–57)	50 (44–54)	0.04*
Female sex	121 (54%)	11 (42%)	110 (56%)	0.19
BMI, kg/m2	22 (21–25)	22 (21–23)	22 (21–25)	0.65
Arterial hypertension	22 (10%)	4 (15%)	18 (9%)	0.32
Dyslipidaemia,	5 (2%)	0 (0%)	5 (3%)	1.00
Diabetes mellitus	44 (20%)	2 (8%)	42 (21%)	0.10
Hyperthyroidism	1 (0%)	1 (4%)	0 (0%)	0.12
Prior stroke	3 (1%)	0 (0%)	3 (2%)	1.00
Pulmonary hypertension	9 (4%)	6 (23%)	3 (2%)	< 0.001*
History of heart failure	8 (4%)	1 (45%)	7 (4%)	0.94
Ferritin, ng/ml	550 (318–883)	440 (249–755)	568 (344–940)	0.08
Pre‐transfusion Hb, g/dl	10 (9.5–10.5)	10 (9.5–10.5)	10 (9.4–10.9)	0.20
Sinus rhythm	223 (100%)	26 (100%)	197 (100%)	
Type of atrial fibrillation				< 0.001*
Paroxysmal	20 (9%)	20 (77%)	0 (0%)	
Persistent	6 (3%)	6 (23%)	0 (0%)	
**Cardiovascular drugs**				
Aspirin	84 (38%)	76 (39%)	8 (31%)	0.44
Diuretics	44 (20%)	33 (17%)	11 (42%)	0.002*
ACE‐I or ARBs	42 (19%)	32 (16%)	10 (38%)	0.007*
Anticoagulants				< 0.001*
NOACs	19 (9%)	7 (4%)	12 (46%)	
VKA	8 (4%)	2 (1%)	6 (23%)	
Antiarrhythmic drugs				< 0.001*
Flecainide	1 (0%)	0 (0%)	1 (4%)	
Amiodarone	5 (2%)	0 (0%)	5 (19%)	
Beta‐blockers	42 (19%)	27 (14%)	15 (58%)	
**Echo characteristics**				
LV EDV indexed, ml/m2	57 (48–67)	59 (51–65)	57 (48–67)	0.59
LVEF, %	61 (5)	59 (5)	62 (5)	0.01*
LA ESV indexed, ml/m2	31 (24–39)	41 (33–47)	29 (23–37)	< 0.001*
RA ESV indexed, ml/m2	24 (19–32)	30 (23–40)	24 (19–30)	0.001*
E/A ratio	1.2 (1.0–1.5)	1.4 (1.1–1.9)	1.2 (1.0–1.5)	0.13
E/e' ratio	7.6 (6.2–9.0)	7.9 (7.0–9.5)	7.5 (6.1–8.9)	0.21
sPASP, mmHg	26 (22–30)	30 (28–33)	25 (21–30)	0.001*
RV EDA indexed, cm2/m2	11 (10–13)	11 (10–12)	12 (10–13)	0.19
TAPSE, cm	2 (2–3)	2 (2–3)	2 (2–3)	0.47
Severe mitral or aortic valve disease	0 (0%)	0 (0%)	0 (0%)	
**CMR characteristics**				
LVEF, %	63 (60–67)	63 (57–68)	63 (60–67)	0.99
EDM indexed, g/m2	50 (13)	56 (12)	49 (13)	0.01*
RVEF, %	63 (7)	62 (8)	63 (6)	0.70
Native septal T1 mapping	955 (914–987)	935 (909–980)	955 (920–987)	0.47
Septal T2*	38 (35–41)	38 (36–42)	38 (35–41)	0.34
Liver T2*	9 (4–18)	9 (4–21)	9 (4–18)	0.62

Data are expressed as mean (SD) or median (IQR).

Abbreviations: ACE‐I: ace‐inhibitors; ARBs: angiotensin receptor blockers; AF: atrial fibrillation; BMI: body mass index, EDA: end‐diastolic area; EDM: end‐diastolic mass; EDV: end‐diastolic volume; ESV: end‐systolic volume; Hb: haemoglobin; LA: left atrium; LV: left ventricle; LVEF: left ventricular ejection fraction; NOACs: Non‐vitamin K antagonist oral anticoagulants; RA: right atrium; RV: right ventricle; RVEF: right ventricular ejection fraction; sPASP: pulmonary artery systolic pressure; TAPSE: tricuspid annular systolic excursion; VKA: vitamin K antagonists.

### 3.2 Primary Endpoint

**FIGURE 2:**
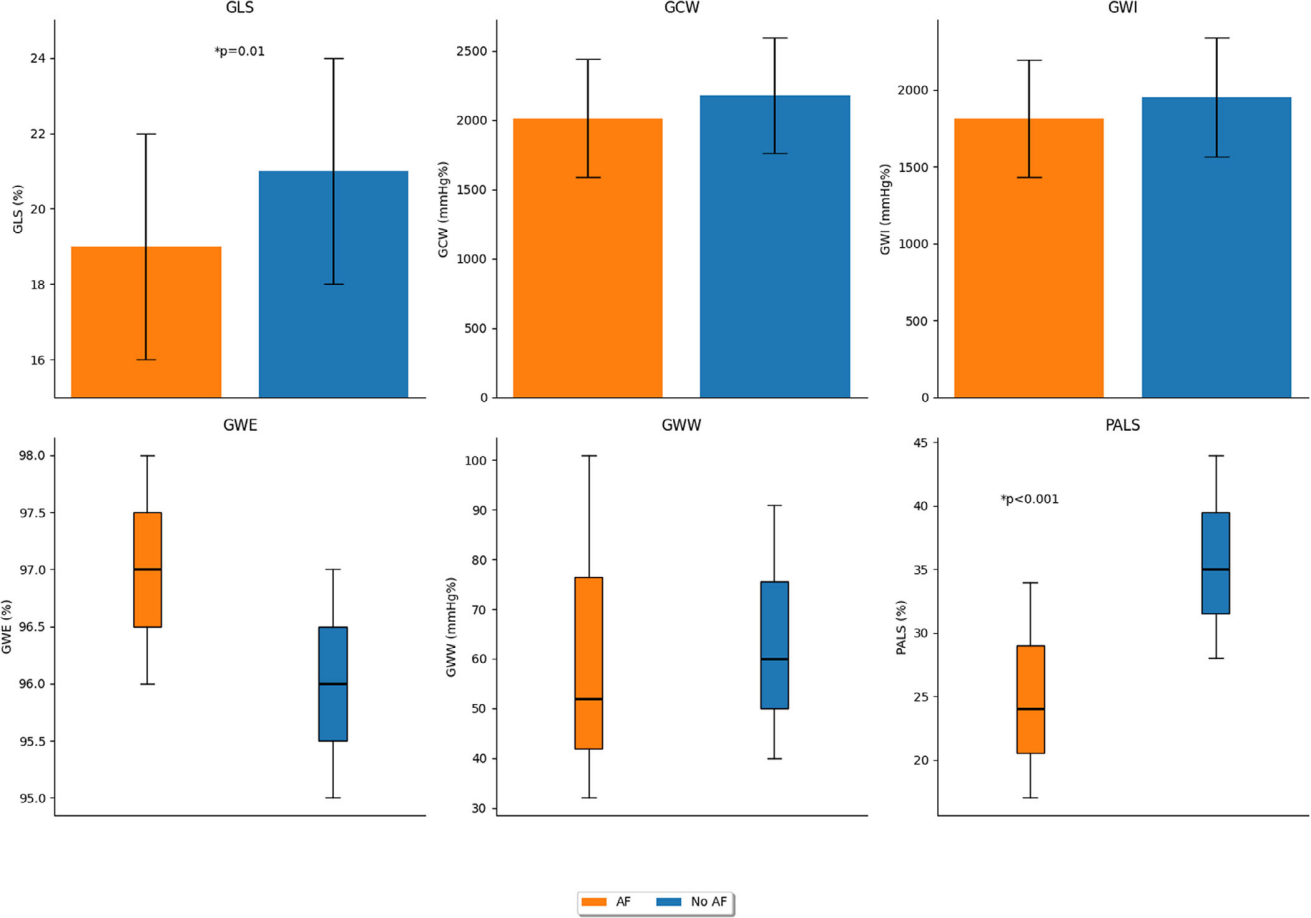
STE indices between patients with and without AF. GLS: global longitudinal strain (reported as absolute values); GCW: global constructive work; GWI: global work index; GWW: global wasted work; GWE: global work efficiency, PALS: peak atrial longitudinal strain. AF: atrial fibrillation.

Patients with history of AF showed significantly lower values of GLS (19% vs 21%, *p* = 0.01) compared to those without AF. Additionally, PALS was markedly lower in AF group than non‐AF group (24% vs 35%, *p* < 0.001). No differences were found in MW parameters between the study groups (Table 2, Figure 2).

**TABLE 2:** Difference in indices of cardiac mechanics between the study groups.

	Total	History of AF	No AF	
	*N* = 223	*N* = 26	*N* = 197	*p*‐value
GLS, %	21 (3)	19 (3)	21 (3)	0.01*
GCW, mmHg%	2159 (419)	2014 (426)	2177 (415)	0.07
GWI, mmHg%	1933 (388)	1814 (380)	1949 (387)	0.11
GWW, mmHg%	60 (40–92)	52 (32–101)	60 (40–91)	0.55
GWE, %	96 (95–97)	97 (96–98)	96 (95–97)	0.49
PALS, %	35 (26–44)	24 (17–34)	35 (28–44)	< 0.001*

Data are expressed as mean (SD) or median (IQR).

Abbreviations: AF: atrial fibrillation; GLS: global longitudinal strain (reported as absolute values); GCW: global constructive work; GWI: global work index; GWW: global wasted work; GWE: global work efficiency, PALS: peak atrial longitudinal strain.

### 3.3 Secondary Endpoint

**FIGURE 3:**
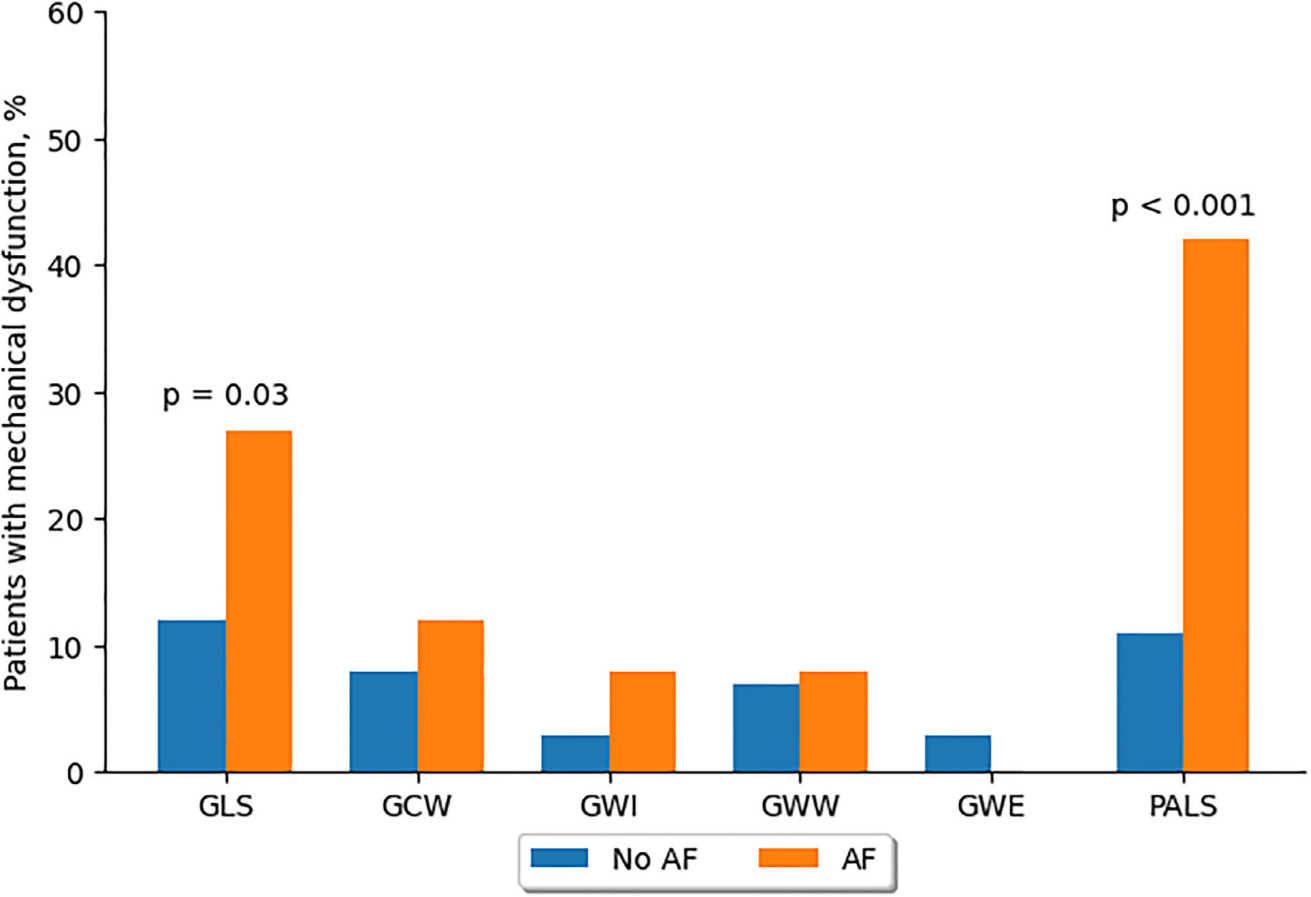
Prevalence of mechanical dysfunction between patients with and without history of AF. GLS: global longitudinal strain; GCW: global constructive work; GWI: global work index; GWW: global wasted work; GWE: global work efficiency, PALS: peak atrial longitudinal strain. AF: atrial fibrillation.

Reduced GLS values were observed in 7 patients with history of AF (27%) and in 23 patients without AF (12%) (*p* = 0.03). No significant differences were found between AF and non‐AF groups in the percentage of patients with reduced MW indices. In contrast, a significantly higher proportion of patients with history of AF showed reduced PALS values compared to those without AF (42% vs 11%, *p* < 0.001) (Table 3, Figure 3)

**TABLE 3:** Proportion of patients with reduced STE indices in the study groups.

	Total	History of AF	No AF	
	*N* = 223	*N* = 26	*N* = 197	*p*‐value
GLS, n%	30 (13%)	7 (27%)	23 (12%)	0.03*
GCW, n%	18 (8%)	3 (12%)	15 (8%)	0.49
GWI, n%	8 (4%)	2 (8%)	6 (3%)	0.23
GWW, n%	15 (7%)	2 (8%)	13 (7%)	0.83
GWE, n%	5 (2%)	0 (0%)	5 (3%)	0.41
PALS, n%	33 (15%)	11 (42%)	22 (11%)	< 0.001*

Abbreviations: AF: atrial fibrillation; GLS: global longitudinal strain; GCW: global constructive work; GWI: global work index; GWW: global wasted work; GWE: global work efficiency, PALS: peak atrial longitudinal strain.

**TABLE 4:** Univariate and multivariate logistic regression analysis for atrial fibrillation.

	OR	95% CI	*p*‐value
***Univariate analysis***			
Age	1.04	1.01–1.09	0.03*
sPASP	1.09	1.04–1.16	0.001*
Septal T2*	1.00	0.95–1.05	0.78
LA ESV indexed	1.07	1.04–1.10	< 0.001*
GLS < 18.9%	3.11	1.27 –7.57	0.01*
PALS < 25.9%	10.35	4.44 – 24.13	< 0.001*
**Multivariate analysis**			
**Model 1**			
Age	0.97	0.91–1.04	0.45
sPASP	1.07	1.01–1.13	0.01*
LA ESV indexed	1.03	1.00–1.07	0.02*
GLS < 18.9%	3.72	1.23–11.23	0.01*
**Model 2**			
Age	0.94	0.88–1.01	0.11
sPASP	1.06	1.00 – 1.13	0.03*
LA ESV indexed	1.01	0.98 – 1.05	0.27
PALS < 25.9%	4.85	1.44 – 16.33	0.01*

Abbreviations: GLS: global longitudinal strain (absolute values); LA ESV: left atrial end systolic volume; PALS: peak atrial longitudinal strain; sPASP: pulmonary artery systolic pressure.

### 3.4 ROC Curve Analysis

**FIGURE 4:**
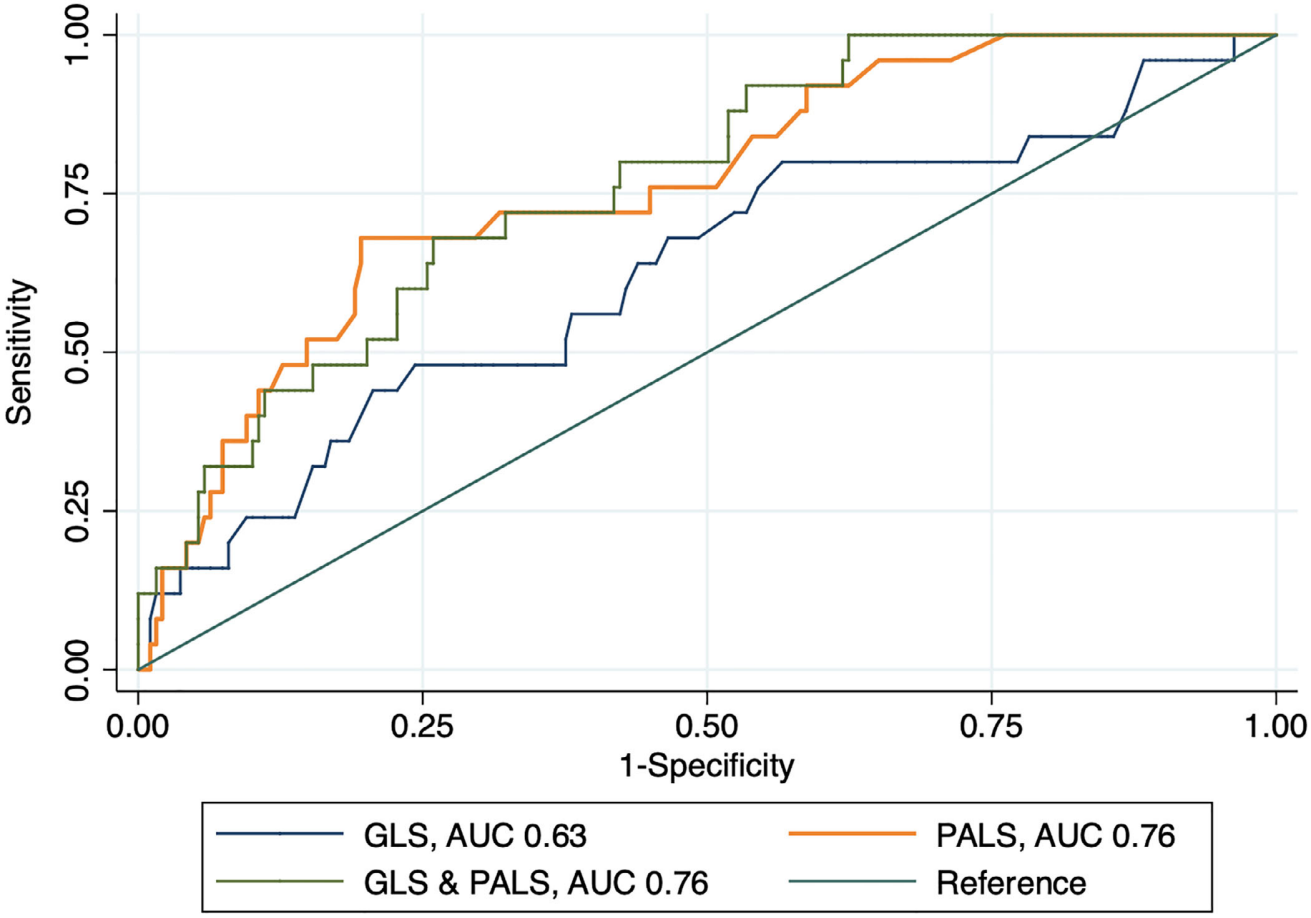
ROC curve analysis for diagnostic accuracy of STE indices. GLS: global longitudinal strain (absolute values); PALS: peak atrial longitudinal strain; AUC: area under the curve.

GLS provided low discriminatory accuracy for AF (AUC 0.63, 95% CI 0.51–0.75) and the optimal cut‐off value was identified at 18.9%; at this cut point, the sensitivity was 50% and the specificity was 75%. PALS showed high discriminative ability (AUC 0.76, 95% CI 0.66–0.85); the optimal cut‐off value was determined to be 25.9%, with a sensitivity of 68% and a specificity of 80%. (Figure 4).

### 3.5 Uni and Multivariate Analysis

Univariate logistic regression analyses showed that age, sPASP, LA ESV indexed, GLS < 18.9% and PALS < 25.9% were associated with history of AF. After multivariate adjustment, the independent association with history of AF was confirmed for sPASP, LA ESV indexed and absolute GLS < 18.9% with an OR for GLS of 3.72 (95% CI 1.23 – 11.23, *p* = 0.01) in model 1. In model 2, sPASP and PALS less than 25.9% remained independent associated with history of AF, with PALS < 25.9% corresponding to an OR of 4.85 (95% CI 1.44–16.33, *p* = 0.01) (Table 4).

## 4 Discussion

The main findings of the present study are the following:

1. In patients with TDT and history of AF, both GLS and PALS values were significantly lower and independently associated to history of AF.
2. TDT patients with history of AF displayed higher prevalence of both ventricular and atrial mechanical dysfunction compared to non‐AF patients.

Since the introduction of blood transfusions and iron chelation therapy, the survival of TDT patients has radically improved, leading to a life expectancy almost comparable to the one of general population [14, 15]. Refractory heart failure and life‐threatening ventricular arrhythmias have given way to new comorbidities, such as supraventricular arrhythmias and, especially, AF [16, 17, 18]. The prevalence of AF in TDT is higher than general population and it can develop even in patients with an optimal chelation status according to T2* values [5]. Iron overload, in fact, is the main risk factor for the development of AF; however, the extensive use of iron chelation therapy has led to an increasing number of patients becoming free from iron overload. Therefore, new markers are needed for early identification of AF. In addition to the traditional risk factors for AF, several authors have directed their attention towards electrocardiographic and echocardiographic parameters for the evaluation of atrial function. Russo et al. found that a P wave dispersion > 35.5 ms had a sensitivity of 90% and a specificity of 85% for identification of AF, while maximum P wave duration > 111 ms displayed a sensitivity of 80% and a specificity of 87% [19]. Rago and colleagues demonstrated an association between atrial electromechanical delay (AEMD) and the occurrence of AF, identifying threshold values of 40.1 ms for intra‐left AEMD and 44.8 ms for inter‐AEMD. These cut‐off points corresponded to sensitivities of 76.2% and 81.2%, and specificities of 97.5% and 98.7%, respectively [20]. Other known risk factors are left atrial dilation identified by CMR and supraventricular ectopic beats burden, which may create the substrate for the development of AF [21, 22].

Recently, STE has emerged as a valuable tool for the assessment of myocardial deformation in various CV diseases. Specifically, patients suffering from AF display lower PALS values, reflecting impaired atrial function [23]. Furthermore, PALS has been identified as a strong predictor not only for the development of AF but also for recurrence of the arrhythmia following electrical cardioversion or catheter ablation [24].

Several works assessed the association between GLS and myocardial iron overload, however, the potential role of STE in evaluating cardiac function in patients with TDT with AF has not been completely investigated. In the work of Patsourakos et al., TDT patients with AF had lower values of GLS and PALS than both TDT patients without AF and controls. Similarly, patients with myocardial iron overload had lower values of both GLS and PALS, suggesting that STE could represent a solid tool to early identify TDT patients at risk for the development of AF [25].

To the best of our knowledge, this is the first study to conduct a complete assessment of cardiac mechanics through the evaluation of all STE indices in TDT patients with history of AF. We found that patients with history of AF, even if in sinus rhythm, showed lower values of both GLS and PALS, reflecting reduced longitudinal ventricular deformation and atrial function in this group of patients.

Notably, these parameters remained independently associated to history of AF even after multivariate adjustment, highlighting their potential as robust markers of subclinical cardiac dysfunction. In contrast, no significant differences were observed in MW parameters between patients with or without AF. This finding likely reflects the relatively preserved global LV systolic function and similar loading conditions in both groups, as well as the fact that MW primarily captures global systolic performance rather than subtle longitudinal abnormalities. In contrast, GLS and particularly PALS appear to detect earlier stages of myocardial and atrial dysfunction that are not yet accompanied by a reduction in MW indices [8].

Specifically, our results identified the left atrium as the most affected cardiac chamber. In fact, not only patients with history of AF had significantly lower PALS values compared to those without AF, but more than 40% of them had values below the normal reference cut‐off, suggesting a deterioration in atrial reservoir function associated with the arrhythmia. Moreover, the ROC analysis for PALS demonstrated high diagnostic performance, with an AUC of 0.76, reflecting a good ability to distinguish between patients with and without history ofAF. Conversely, in our cohort of TDT patients, global right ventricular (RV) systolic function was preserved on CMR and longitudinal RV function by conventional echocardiography (e.g., TAPSE) was also preserved and comparable between groups, supporting the interpretation that the mechanical alterations associated with AF are more consistent with atrial and left‐sided subclinical dysfunction rather than overt RV systolic impairment.

The findings of our study carry clinically relevant messages for the management of TDT population. In fact, AF relapses are particularly symptomatic in this cohort patients, leading to high rate of hospital admissions [5] with a relevant impact on quality of life. An early identification of impaired mechanical function by STE (mainly PALS and GLS) may lead to a close monitoring of those at high risk of relapses or progression to permanent AF. In daily practice, this could translate into closer rhythm surveillance, earlier referral to electrophysiological evaluation when appropriate, and a lower threshold for implementing rhythm control strategies once AF is documented. Moreover, patients with reduced STE indices might benefit from a more intensive optimization of iron chelation therapy, careful management of volume status and tighter control of CV risk factors.

These findings are also important considering that these patients present an excellent chelation status according to T2*, indicating that traditional iron overload indices alone are not sufficient and that novel markers are required for the early identification of mechanical dysfunction. These results support the hypothesis that abnormalities in myocardial mechanics, detected through STE, may serve as sensitive markers of AF in TDT patients. Future prospective studies, however, are needed to confirm these hypotheses.

## 5 Limitations

This study presents several limitations that should be acknowledged. First, it was conducted as a single‐centre, observational study with a relatively limited sample size, which may affect the generalisability of the findings to broader populations. Second, the presented data are cross‐sectional and further multicentre prospective studies with larger sample size are warranted to confirm and expand upon these findings. Third, the exclusion of patients with inadequate echocardiographic image quality might have introduced a selection bias, potentially leading to an underestimation of the prevalence of cardiac dysfunction. Fourth, a dedicated assessment of RV free‐wall mechanics, including RV free‐wall strain, was not performed; therefore, subtle regional RV dysfunction cannot be excluded, even though CMR RVEF and TAPSE were preserved and comparable between groups.

Finally, because the time interval between the echocardiographic exam and blood transfusion was not systematically recorded, transfusion‐related changes in loading conditions may have affected load‐dependent STE indices, particularly GLS and PALS.

## 6 Conclusions

Among TDT patients, those with history of AF showed lower values of GLS and PALS. Both LV and atrial mechanical dysfunction were significantly prevalent in patients with history of AF and PALS showed high diagnostic accuracy for the detection of AF.

## Funding

The authors declare that no funds, grants, or other support were received during the preparation of this manuscript.

## Disclosures

The authors have nothing to report.

## Ethics Statement

β‐THAL study was approved by the local Ethics Committee (Comitato Etico Indipendente di Area Vasta Emilia Centro).

## Consent

All participants gave consent for participation in the β‐THAL study

## Conflicts of Interest

The authors have no relevant financial or non‐financial interests to disclose.

## Supporting information



Supporting File 1: echo70408‐sup‐0001‐SuppMat.docx

## Data Availability

All data relevant to the study are included in the article or uploaded as supporting information.
