# Supplementary material for: Assessing Cardiac Mechanical Dysfunction in Transfusion‐Dependent β‐Thalassemia With History of Atrial Fibrillation: The Role of Speckle Tracking Echocardiography
Source: Echocardiography. 2026 Feb 11;43(2):e70408. doi: 10.1111/echo.70408 (PMC12895093; doi:10.1111/echo.70408)
Supplement: Supplementary file 1 — Supporting File 1: echo70408‐sup‐0001‐SuppMat.docx [file ECHO-43-e70408-s001.docx]

**Supplementary Data**

**Assessing cardiac mechanical dysfunction in transfusion-dependent β- thalassemia with history of atrial fibrillation: the role of speckle tracking echocardiography**

- **Detailed echocardiography protocol page 2**
- **STE normal values page 3**

**Detailed echocardiography protocol**

**Acquisition**

Left ventricular (LV) end-diastolic and end-systolic volumes were obtained from both the four-chamber (4CH) and two-chamber (2CH) apical views using the biplane Simpson’s method. The left ventricular ejection fraction (LVEF) was then derived using the standard biplane formula. Assessment of cardiac valvular regurgitations was conducted according to the latest EACVI recommendations^1^. The mitral flow pattern was measured using pulsed wave (PW) Doppler by placing the sample volume at the mitral valve leaflet tips in the apical 4CH view.  E’ velocity was measured using pulsed-wave tissue Doppler imaging (TDI) by placing the sample volume at the septal and lateral mitral annulus in the apical 4CH. Tricuspid annular plane systolic excursion (TAPSE) was determined based on the M-mode recording. The right ventricular end-diastolic area was measured at end-diastole from the apical 4CH view. For two-dimensional speckle-tracking echocardiography (2D-STE), the imaging sector’s size and depth were modified to ensure full visualization of the LV myocardium across all three apical views. Frame rates were maintained within the 40–80 frames per second (fps) range to optimize temporal resolution^2^.

**Strain and myocardial work post-processing**

End-systole was identified using the timing of aortic valve closure in the apical long-axis view. Regions of interest (ROIs) were carefully traced along myocardial boundaries at end-systole in each apical view. Longitudinal strain was calculated for the entire myocardium in each LV apical window and reported as a color-coded polar plot (bull’s eye)^2^. A standardized 17-segment LV model was employed for analysis of longitudinal strain. Peak atrial longitudinal strain (PALS) was calculated in both 4CH and 2CH views, by outlining the atrial borders, and the average value was reported^3^.

Myocardial work quantification relied on pressure–strain loop (PSL) analysis. Timing of valvular events was determined from pulsed-wave Doppler recordings. The following myocardial work parameters were derived: global work index (GWI - area under the loop), global constructive work (GCW), global wasted work (GWW), and global work efficiency (GWE)^4^.

**Normal values used for STE indices**

GLS (absolute values): < 17.2%^5^

PALS: > 23%^6^

Global work index: > 1292 mmHg%^4^

Global constructive work: > 1582 mmHg%^4^

Global wasted work: < 254 mmHg%^4^

Global work efficiency: > 90%^4^

**REFERENCES**

1. Lancellotti P, Pibarot P, Chambers J, Canna G La, Pepi M, Dulgheru R, Dweck M, Delgado V, Garbi M, Vannan MA, Montaigne D, Badano L, Maurovich-Horvat P, Pontone G, Vahanian A, Donal E, Cosyns B. Multi-modality imaging assessment of native valvular regurgitation: an EACVI and ESC council of valvular heart disease position paper. *Eur Heart J Cardiovasc Imaging* 2022;23:e171–e232.

2. Voigt J-U, Pedrizzetti G, Lysyansky P, Marwick TH, Houle H, Baumann R, Pedri S, Ito Y, Abe Y, Metz S, Song JH, Hamilton J, Sengupta PP, Kolias TJ, d’Hooge J, Aurigemma GP, Thomas JD, Badano LP. Definitions for a common standard for 2D speckle tracking echocardiography: consensus document of the EACVI/ASE/Industry Task Force to standardize deformation imaging. *Eur Heart J Cardiovasc Imaging* 2015;16:1–11.

3. Pathan F, D’Elia N, Nolan MT, Marwick TH, Negishi K. Normal Ranges of Left Atrial Strain by Speckle-Tracking Echocardiography: A Systematic Review and Meta-Analysis. *Journal of the American Society of Echocardiography* 2017;30:59-70.e8.

4. Manganaro R, Marchetta S, Dulgheru R, Ilardi F, Sugimoto T, Robinet S, Cimino S, Go YY, Bernard A, Kacharava G, Athanassopoulos GD, Barone D, Baroni M, Cardim N, Hagendorff A, Hristova K, López-Fernández T, la Morena G de, Popescu BA, Penicka M, Ozyigit T, Rodrigo Carbonero JD, Veire N van de, Bardeleben RS Von, Vinereanu D, Zamorano JL, Rosca M, Calin A, Moonen M, Magne J, Cosyns B, Galli E, Donal E, Carerj S, Zito C, Santoro C, Galderisi M, Badano LP, Lang RM, Oury C, Lancellotti P. Echocardiographic reference ranges for normal non-invasive myocardial work indices: results from the EACVI NORRE study. *Eur Heart J Cardiovasc Imaging* 2019;20:582–590.

5. Sugimoto T, Dulgheru R, Bernard A, Ilardi F, Contu L, Addetia K, Caballero L, Akhaladze N, Athanassopoulos GD, Barone D, Baroni M, Cardim N, Hagendorff A, Hristova K, Lopez T, la Morena G de, Popescu BA, Moonen M, Penicka M, Ozyigit T, Rodrigo Carbonero JD, Veire N van de, Bardeleben RS von, Vinereanu D, Zamorano JL, Go YY, Rosca M, Calin A, Magne J, Cosyns B, Marchetta S, Donal E, Habib G, Galderisi M, Badano LP, Lang RM, Lancellotti P. Echocardiographic reference ranges for normal left ventricular 2D strain: results from the EACVI NORRE study. *Eur Heart J Cardiovasc Imaging* 2017;18:833–840.

6. Nielsen AB, Skaarup KG, Hauser R, Johansen ND, Lassen MCH, Jensen GB, Schnohr P, Møgelvang R, Biering-Sørensen T. Normal values and reference ranges for left atrial strain by speckle-tracking echocardiography: the Copenhagen City Heart Study. *Eur Heart J Cardiovasc Imaging* 2021;23:42–51.
